# Association of Preoperative Plasma D-dimmer and Fibrinogen and Renal Cell Carcinoma Outcome

**DOI:** 10.7150/jca.31173

**Published:** 2019-07-10

**Authors:** Xiaobo He, Tao Huang, Yunfei Xue, Meng Zhang, Qiaodan Liu, Yongqiang Wang, Kai Yao, Shengjie Guo

**Affiliations:** 1 Department of Urology, Sun Yat-Sen University Cancer Center, State Key Laboratory of Oncology in South China, Collaborative Innovation Center for Cancer Medicine, Guangzhou, China.; 2 Department of Medical Oncology, the Fifth Affiliated Hospital of Sun Yat-Sen University, Zhuhai, China.; 3 Department of Urology, Shunde People's Hospital, Southern Medical University, Guangdong, China.; 4 Vascular Surgery Center, Fuwai Hospital, Chinese Academy of Medicine Sciences Beijing, China.; 5 B.M. Urinary Surgery, China-Japan Friendship Hospital, Beijing, China.; 6 Department of Biochemistry and Molecular Medicine, school of Medicine, University of California Davis, Sacramento, California, USA.

**Keywords:** renal cell cancer, coagulation-fibrinolysis system, fibrinogen, D-dimer, prognosis.

## Abstract

**Background**: the plasma D-dimer and fibrinogen which are indicators of coagulation-fibrinolysis system has been reported to be associated with survival in several types of cancers, including RCC. The aim of our study was to assess the prognostic significance of preoperative plasma D-dimer and fibrinogen levels in RCC patients.

**Methods**: Data from 449 patients with RCC were assessed retrospectively. Cutoff value for plasma D-dimer and fibrinogen were tested by the standardized cutoff-finder algorithm. Overall survival (OS) and disease-free survival (DFS) were evaluated using the Kaplan-Meier method. Univariate and Multivariate Cox regression models were further applied for two end points.

**Results**: Multivariate analysis identified increased plasma D-dimer and fibrinogen as independent prognostic factors for OS (D-dimer, P=0.017; Fibrinogen, P=0.049) and DFS (D-dimer, P=0.038; Fibrinogen, P<0.001). Moreover, all the patients were stratified using these two factors in the following ways: (1) Low risk: both level of plasma D-dimer and fibrinogen were no more than cutoff value. (2) Intermediate risk: neither low risk nor high risk, (3) high risk: both level of plasma D-dimer and fibrinogen were higher than cutoff value. This model showed significant predictive power for OS and DFS.

**Conclusion**: preoperatively elevated D-dimer and fibrinogen can be regard as independent predictors for patients' prognosis in RCC. Combining both plasma D-dimer and fibrinogen can improve the prognostic accuracy and easy accessibility in clinical practice.

## Introduction

Renal cell carcinoma (RCC) comprises approximately 1.6% of all new malignancies in Chinese adults for the most recent 3 years 1, and these are primarily renal cell carcinomas, also called renal adenocarcinoma, which occur in the body of the kidney. Kidney cancer incidence rates increased over the past several decades, in part due to incidental diagnoses during abdominal imaging 2. Surgery, including traditional and laparoscopic (i.e. minimally invasive, performed through very small incisions), is the primary treatment for most RCC patients 3. Renal cell cancer can often be cured if it is diagnosed and treated when still localized to the kidney and to the immediately surrounding tissue 4. In addition, the application of prognostic factors can accurately predict prognosis of RCC patients are of dominant interest, not only for patients' individualized risk evaluation but also for the comparison of the results from international clinical multicenter trials 5.

It is evident that the plasminogen-plasmin and coagulation system is a vital component in the biology of neoplastic disease 6, 7. According the existing literatures, the two parts (fibrinogen and D-dimer) of the plasminogen-plasmin and coagulation system may be related to prognosis in RCC.1) Fibrinogen, a glycoprotein synthesized by hepatocytes with the function of blood coagulation, namely the clotting factors. It is a target molecule at the final step of the coagulation cascade that is converted to fibrin monomers by thrombin and ultimately forms fibrin polymers 8. A study shows that fibrinogen increases the metastatic potential of circulating tumor cells 9. And another study shows that preoperative plasma fibrinogen level can be a significant prognostic factor in patients with renal cell carcinoma after surgical treatment 10. 2) D-dimer is a specific degradation product, after fibrin monomer was crosslinked by activation factor XIII and then was hydrolyzed by fibrinolytic enzyme. D-dimer is a biomarker of specific fibrinolytic process. It is from a kind of crosslinked fibrin clot, what is dissolved by fibrinolytic enzymes. Nowadays, D-dimer has been deemed to a potential biomarker for many diseases 11, 12.

As far as we known, the plasminogen-plasmin and coagulation system are studied infrequently in RCC. In our present study, we retrospectively evaluated the relationships between the fibrinogen, D-dimer and patient survival. In the same time, we sought to determine whether preoperative fibrinogen and D-dimer are prognostic factors. Specially, we stratified all the patients based on these two factors into three cohorts and studied whether the three cohorts have different prognosis or not.

## Methods

### Patients

This retrospective analysis included data from 912 consecutive RCC patients who underwent a curative radical or partial nephrectomy at the department of urology in sun yat-sen university cancer center (SYSUCC), Between January 2000 and December 2012. The following items are the inclusion criteria for this research: (i) diagnosed as RCC pathologically; (ii) access to complete clinical data and preoperative blood sampling for D-dimer and fibrinogen levels; (iii) effective and accurate follow-up. Of this data set, the 449 patients were included in our study. All the clinicopathological data were retrieved from the electronic patient records of our center. Informed consent was waived because of the retrospective research of the study and the analysis used anonymous clinical data.

### Patients Follow-up

Follow-up was carried out by telephone interview and complimentary medical records review. Important follow-up data included postoperative adjuvant therapy, living status, progression and sites of tumor metastases. The last follow-up was completed in November 01, 2015, and after that, the whole data were analyzed. The primary endpoint was overall survival (OS) which defined that the interval between surgery and last follow-up or death. Secondary endpoint was disease free survival (DFS) which calculated the interval between surgery and last follow-up or recurrence or death.

### Statistical analysis

Continuous variables were presented as mean and standard deviations and categorical variables were expressed as frequencies and percentages. To evaluate the best cutoff points of D-dimer and fibrinogen to predict prognosis, we used a validated web-based software 13. In short, the variable was dichotomized at each possible cutoff point, and Cox proportional hazard models were applied to the variables measured and the survival variable. Survival analysis was executed using the R package “survival.” According to the overall survival status, the optimal cutoff was defined as the point which gave the most significant log-rank cohort split. OS and DFS after surgery were measured by using of Kaplan Meier curves and the log-rank test. Univariate Cox regression analyses were done to compare all the variables and significant prognostic factors identified from the univariate analysis were entered the multivariate Cox regression analyses of survival to test for independence. Hazard ratios (HRs) estimated from the Cox analysis was reported as relative risks with corresponding 95% confidence intervals (CIs). All statistical analyses were performed using SPSS21.0 software (IBM, Armonk, NY) and EmpowerStats software (www.empowerstats.com, X&Y solutions, Inc. Boston MA). All tests were two-sided and a P value <0.05 was considered statistically significant.

## Results

A total of 449 patients with RCC were enrolled in our study which comprised 292 males and 157 females at a mean age of 52.06 years (SD: ±13.49). 361 (80.4%) had clear cell, 28 (6.2%) had papillary 23 (5.2%) had chromophores RCC and 37 (8.2%) were not otherwise specified. According to the TNM staging system, 314 (69.9%), 67 (14.9%), 43 (9.6%) and 25 (5.6%) were staged in Ⅰ, Ⅱ, Ⅲ and Ⅳ, separately. The demographic and clinicopathologic parameters of the study cohort are shown in **Table 1**.

**Figure 1:**
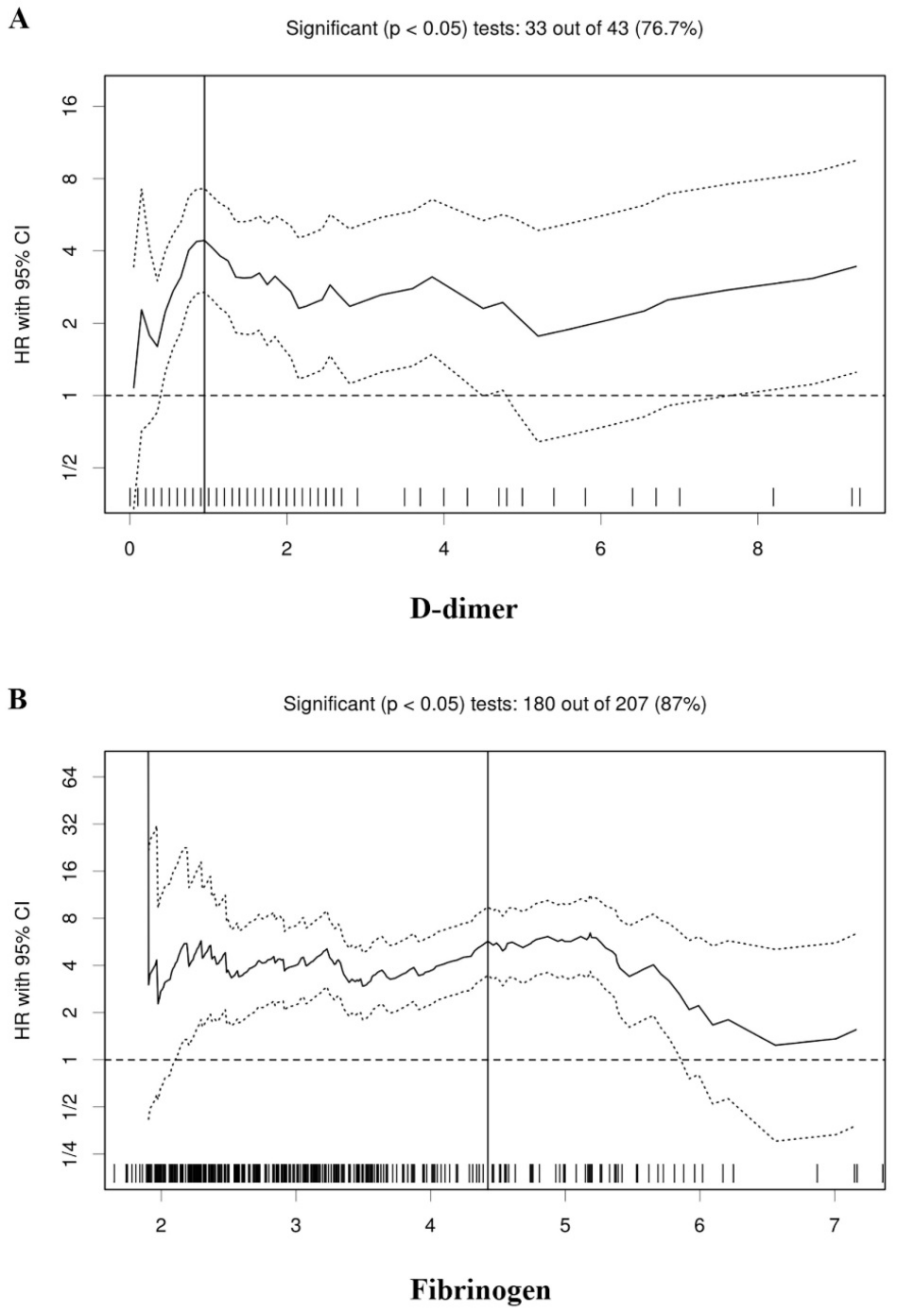
Hazard ratio (HR) for overall survival in dependence of cutoff points for plasma D-dimer (A) and fibrinogen (B). A vertical line designates the chosen cutoff point. The plots were generated using the biostatistical tool, cutoff finder.

According to the Cutoff Finder, the optimal cutoff values of the D-dimer and fibrinogen were determined to be 0.95 and 4.42 for the prognosis of RCC patients, respectively (**Figure 1**). According to the cutoff values, all the patients were divided into the low and high groups, respectively (**Table 1**).

**Figure 2:**
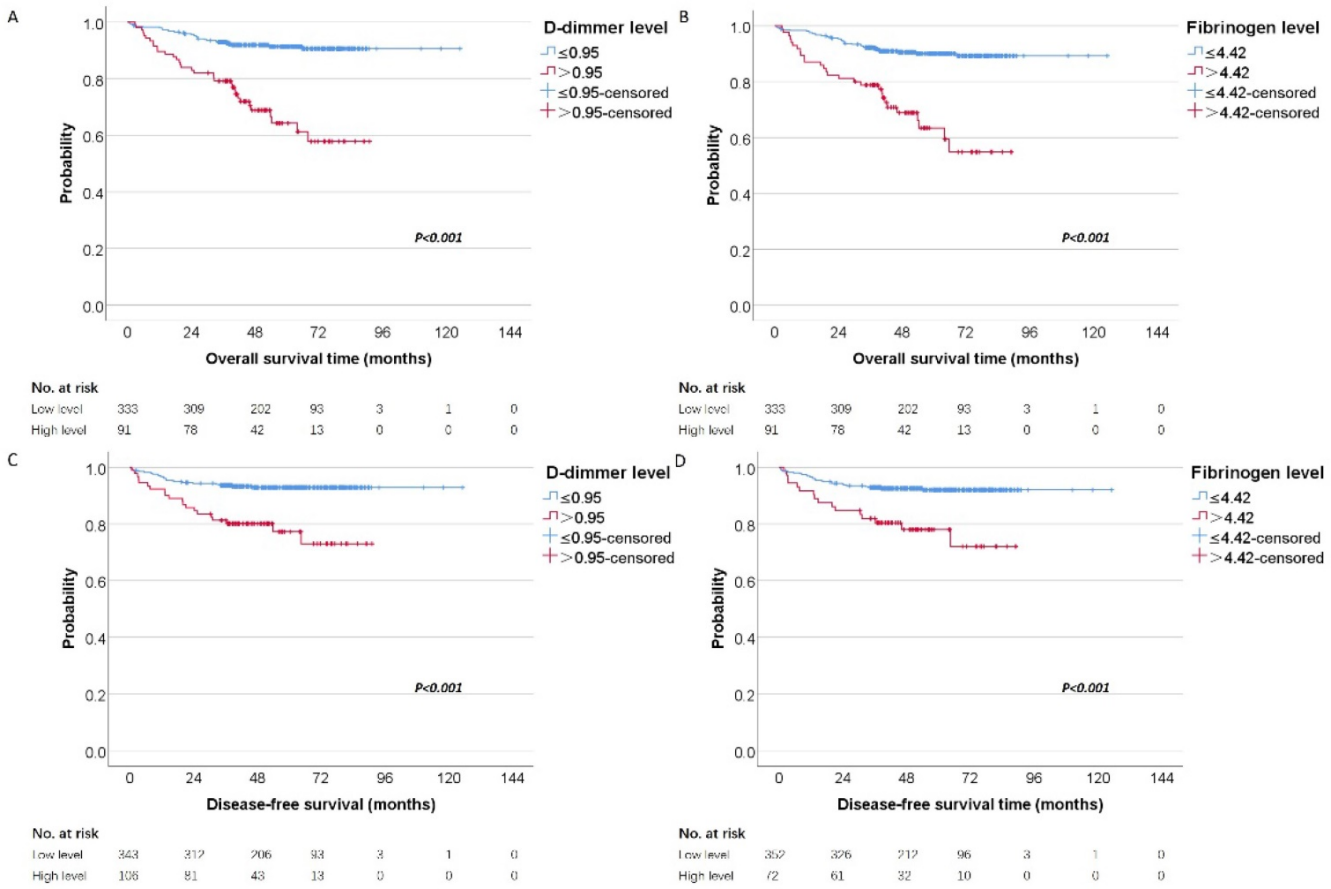
Kaplan-Meier estimates of overall survival stratified by the chosen cutoff points of D-dimer (A) and fibrinogen (B), respectively. Kaplan-Meier estimates of disease-free survival stratified by the chosen cutoff points of D-dimer (C) and fibrinogen (D), respectively.

To investigate whether the D-dimer and fibrinogen were related to the clinical outcome of RCC patients, univariate and multivariate analyses for OS and DFS were performed. The mean and median follow-up time was 58.71 and 57.72 months. For localized disease, we excluded stage Ⅳ cases resulting in 424 patients with RCC. In our further study, 449 patients were analyzed for OS and 424 were analyzed for DFS. **Figure 2** showed the Kaplan-Meier curves for OS and DFS and reveal that high D-dimer and fibrinogen were consistent factors for poor prognosis in RCC patients (P<0.001 for both two tested end points, log-rank test), respectively.

Univariate analysis identified BMI, blood platelet, pathologic types, Fuhrman grade, pT-stage, pN-stage, pM-stage, clinical stage, D-dimer and fibrinogen were as prognosticators of poor outcome for patients' OS (**Table 2**). In the same way, regarding DFS, univariate analysis revealed that BMI, blood platelet, pathologic types, Fuhrman grade, pT-stage, pN-stage, clinical stage, D-dimer and fibrinogen were associated with prognosis of RCC patients (**Table 3**).

To determine the independent prognostic significance of the D-dimer and fibrinogen for OS and DFS, a multivariate analysis using a Cox proportional hazard model was performed. Regarding OS, in our multivariate analysis that included BMI, blood platelet, pathologic types, Fuhrman grade, pT-stage, pN-stage, pM-stage, D-dimer and fibrinogen, we identified D-dimer (HR=1.47, 95% CI=1.07 to 2.03, P=0.017) and fibrinogen (HR=1.92, 95% CI=1.00 to 3.70, P=0.049) as independent prognostic factors for OS and also including BMI, pT status, pN status and pM status (**Table 2**). For DFS, these clinicopathologic parameters included BMI, blood platelet, pathologic types, Fuhrman grade, pT-stage, pN-stage, D-dimer and fibrinogen brought into our multivariate analysis and we also demonstrated that D-dimer (HR=1.48, 95% CI=1.02 to 2.15, P=0.038) and fibrinogen (HR=3.20, 95% CI=1.62 to 6.34, P<0.001) were independent prognostic factors for DFS (**Table 3**).

**Figure 3:**
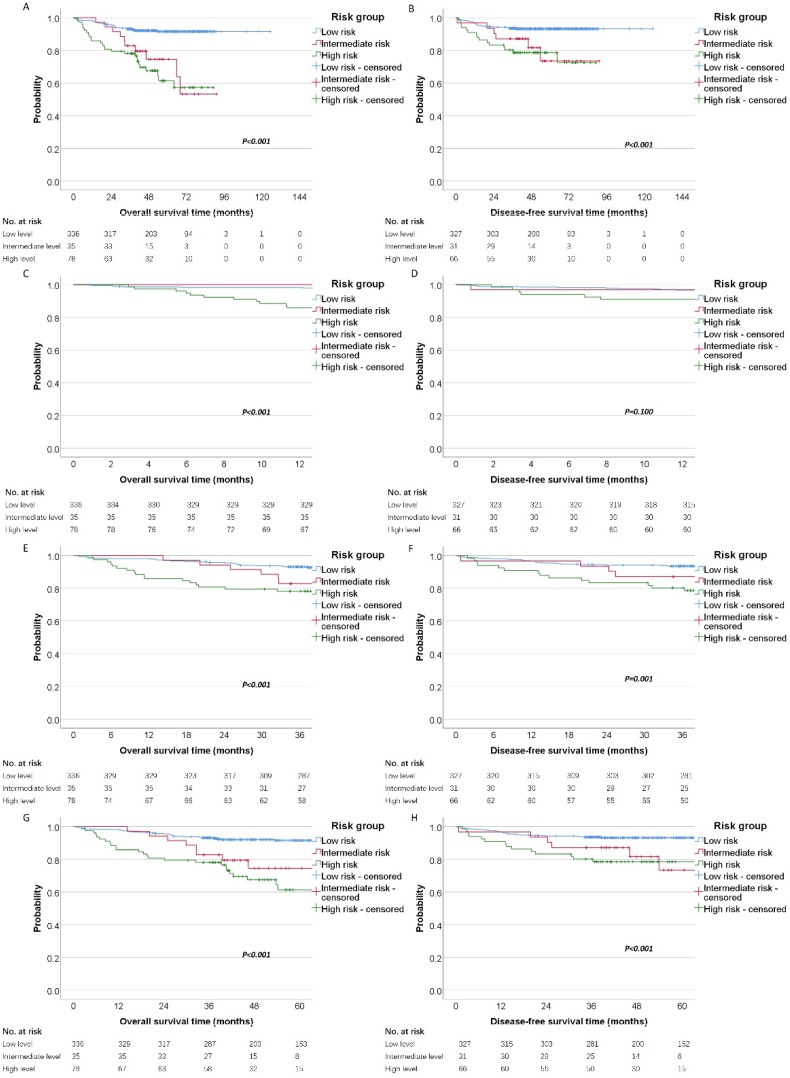
Survival of patients with these three cohorts (low risk, intermediate risk and high risk). Kaplan-Meier estimates of (A) overall survival and disease-free survival (B) according to different risk groups. Meanwhile, the 1-year, 3-year and 5-year survival curves were shown for OS (C, E, G) and DFS (D, F, H).

Finally, patients were categorized into different risk groups according to the level of D-dimer and fibrinogen (Low risk: D-dimer≤0.95 and fibrinogen≤4.42; Intermediate risk: D-dimer≤0.95 and fibrinogen>4.42, or D-dimer>0.95 and fibrinogen≤4.42; High risk: D-dimer>0.95 and fibrinogen>4.42). By the Kaplan-Meier curves and log-rank test, we demonstrated the high risk group had significantly poorer OS (high risk vs. intermediate risk vs. low risk, mean OS time, 116.46 vs 70.82 vs 64.23 months, P < 0.001) and DFS (high risk vs. intermediate risk vs. low risk, mean DFS time, 117.60 vs 76.16 vs 71.77 months, P <0.001) than the other two groups (**Figure 3A and 3B**). Moreover, we analysis the 1-year, 3-years and 5-year survival rate for OS and DFS. The 1-year, 3-year and 5-year OS rate for patients with high risk was 85% (95%CI: 78% to 93%), 78% (95%CI: 69% to 87%) and 61% (95%CI: 49% to 75%) compared with 100%, 82% (95%CI: 71% to 96%), 74% (95%CI: 60% to 92%) for patients with intermediate risk and 98% (95%CI: 96% to 99%), 93% (95%CI: 90% to 95%), 91% (95%CI: 88% to 94%) for patients with low risk. The Kaplan-Meier curves also revealed that the high risk group has the worsen OS than intermediate and low risk for 1-year, 3-year and 5-year survival (**Figure 3C, 3E and 3G**). In the other hand, we also analysis the prognostic significance in these three cohort for DFS. The 1-year, 3-year and 5-year DFS rate for patients with high risk was 90% (95%CI: 84% to 98%), 80% (95%CI: 71% to 90%) and 78% (95%CI: 69% to 89%) compared with 96% (95%CI: 90% to 100%), 87% (95%CI: 76% to 99%), 73% (95%CI: 55% to 97%) for patients with intermediate risk and 96% (95%CI: 94% to 98%), 93% (95%CI: 90% to 96%), 93% (95%CI: 90% to 95%) for patients with low risk. The Kaplan-Meier DFS curve showed that there was significant predictive power for 3-year and 5-year survival (**Figure 3F and 3H**), but not for 1-year survival (**Figure 3D**).

## Discussion

In the present study, we demonstrated that elevated plasma D-dimer or fibrinogen levels were identified as negative prognostic factors for OS and DFS and further research revealed that high and intermediate risk groups were associated with worse OS and PFS of patients with RCC. Despite tremendous development has been made in recent years in terms of genetic, epigenetic and common molecular alterations in RCC 14, 15, pathological examination and traditional clinicopathological prognostic indicators are still the regular diagnostic and prognostic evaluation of RCC. Due to the intricacy of these molecular changes, high costs and the time-consuming preparation, to our best knowledge, little of evidence established how these newly discovered molecular markers influence diagnostic or therapeutic decisions have rendered none of the markers available for routine testing. Regularly measuring blood-based parameters, such as plasma D-dimer and fibrinogen, are rather easy to evaluate without additional laborious efforts for individual risk estimate in preoperative RCC.

Some researchers showed that preoperative plasma fibrinogen independently predicted poor DFS and cancer-specific survival (CSS), whereas D-dimer only had negative independent prognostic value on OS 8. Other study demonstrated preoperative plasma fibrinogen was an independent predictor of distant metastasis and poor prognosis for RCC patients and they did not analyzed the prognostic value of D-dimer levels 16. However, in our study we found elevated D-dimer was associated with poor DFS and OS. The differences in prior studies can be explained by several factors, including the number of patients that 449 patients were enrolled in our study and 128 were in the research of Erdem, S. et al. 8, heterogeneity in the race in the populations studied, the latter being the most critical aspect in calculating the cutoff value. We used the validated web-based software Cutoff Finder which determined a prognostic cutoff point was to optimize the significance of the split in the Kaplan-Meier plot 13.

Fibrinogen is synthesized in the liver, and its level is affected by in vivo infection or inflammation 17. Fibrinogen converted to insoluble fibrin by activated thrombin significantly affects blood clotting, fibrinolysis, inflammatory response, wound healing and neoplasia 18. Pretherapeutic plasma fibrinogen levels have been associated with the clinical outcome of patients with various cancers, including urothelial, colorectal, ovarian, and lung cancer 19-22. The molecular mechanisms underlying the relationships between high plasma fibrinogen and worse survival of patients with RCC have not been fully elucidated 23. However, several possible mechanisms support the observations. This coagulation cascade could be activated by procoagulant activities, which many tumor cells exhibit and that are implicated in the promotion of haematogenous metastasis. Palumbo et al. 9 reported that fibrinogen facilitated tumor stroma formation and promoted the sustained adhesion of circulating tumor cells in the microvasculature, inducing tumor progression and dissemination. Moreover, elevated fibrinogen may protect tumor cell from host's immune defense system. Gunji and Gorelik 24 proposed that fibrin deposition on tumor cells may protect the tumor cell from natural killer cells' cytotoxicity during tumor migration through the blood stream. Subsequently, Zheng et al. 25 also reported that fibrinogen could activate tumor cell adhesion with platelets, forming a dense fibrin layer around the tumor cell that can protect it from the lethal interaction with natural killer cells in the presence of thrombin. Other researchers also suggested that the fibrin matrix may form stromal tissue that provides a nutrient and gas channel for malignant cells 26, 27. For the relationship between D-dimer and tumor progression, the most probable reason is that the abnormal activation of the coagulation-fibrinolysis system. The coagulation-fibrinolysis system is activated abnormally, and it results in promoting tumor growth, invasion, metastasis and angiogenesis 28. Most important of all, abnormal activation of coagulation-fibrinolysis system is reflected by elevated plasma D-dimer level. Our results also support the preoperative elevated D-dimer and fibrinogen levels are as negative prognostic factors for RCC patients.

Our results indicate that the high and intermediate risk may inform frequency of surveillance. Patients with high risk should be close follow-up. Furthermore, according to the different year survival rate for DFS and OS, when the plasma D-dimer or fibrinogen was higher than the cutoff value, the worse trend of survival status was being seen. Given our results and the data from previous basic studies, increased coagulation and fibrinolytic activities appear to be associated with increased risks of tumor progression and metastasis among patients with RCC. Concentrations of D-dimer and fibrinogen before surgery may also offer a useful marker of recurrence and metastasis in RCC patients following resection. Furthermore, functional inhibition of fibrinogen and other coagulation factors might represent novel strategies for treating RCC. Further investigations are needed to clarify the relationships among circulating coagulation and angiogenic factors in neoplastic tissues.

As with all retrospective studies, limitations of our study remain some limitations. Firstly, our outcomes originated from retrospective data. Secondly, the study cohort was small, despite being relatively large compared to other such investigations of RCC patients. Nonetheless, even considering these limitations, our data clearly indicate that increased preoperative D-dimer and fibrinogen might represent independent prognostic factors for OS and DFS in RCC patients.

## Conclusions

In conclusion, preoperatively elevated D-dimer and fibrinogen seems to represent independent predictors with respect to patients' OS and DFS in RCC. Combining both plasma fibrinogen and D-dimer can improve the prognostic accuracy and act as a select criterion for risk factor-stratified patient management in RCC.

## Figures and Tables

**Table 1:** Baseline characteristics of all patients (n=449)

Characteristics	Cases (n=449)	Percentage (%)
**Age (years)**	52.06±13.49	
**BMI**	23.71±3.59	
**Gender**		
Male	292	65.0
Female	157	35.0
**Pathological types**		
Clear cell carcinoma	361	80.4
Papillary carcinoma	28	6.2
Chromophobe renal cell carcinoma	23	5.2
Others	37	8.2
**Fuhrman-grade**		
I	57	12.7
II	219	48.8
III	53	11.8
Ⅳ	5	1.1
Unknown	115	25.6
**pTNM stage**		
I	314	69.9
II	67	14.9
III	43	9.6
Ⅳ	25	5.6
**pT status**		
T1	321	71.5
T2	78	17.3
T3	38	8.5
T4	12	2.7
**pN status**		
N0	419	95.8
N1	30	4.2
**pM status**		
M0	430	95.8
M1	19	4.2
**ALP**		
Low/Normal	435	96.9
Elevated	14	3.1
**Blood platelet**	236.19±73.03	
**Fibrinogen**		
≤4.42	385	85.7
>4.42	64	14.3
**D-dimer**		
≤0.95	343	76.4
>0.95	106	23.6

Abbreviation: BMI, body mass index; pTNM, pathologic tumor-node-metastasis; ALP, alkaline phosphatase.

**Table 2:** Univariate and multivariate analyses for variables considered for overall survival (Cox proportional hazard regression model).

	OS Univariate analysis			OS Multivariate analysis		
Characteristics	95.0% CIs	HR	P value	95.0% CIs	HR	P value
**Age (years)**	1.00 to 1.03	1.01	0.152			
**BMI**	0.78 to 0.91	0.84	<0.001	0.84 to 0.99	0.91	0.036
**Gender**						
Male		1.00(ref.)				
Female	0.69 to 1.92	1.15	0.582	-	-	-
**Pathological types**						
Clear cell carcinoma		1.00(ref.)			1.00(ref.)	
Non-clear cell carcinoma	1.24 to 3.58	2.10	0.006	0.40 to 4.16	1.30	0.655
**Fuhrman-grade**						
I		1.00(ref.)			1.00(ref.)	
II	0.57 to 6.37	1.90	0.298	0.37 to 4.38	1.28	0.692
III	1.61 to 19.85	5.65	0.006	0.50 to 7.27	1.92	0.334
Ⅳ	2.85 to 70.31	14.17	0.001	1.08 to 31.34	5.82	0.040
Unknown	1.28 to 14.18	4.26	0.018	0.22 to 5.42	1.11	0.897
**pTNM stage**						
I		1.0 (ref.)				
II	0.98 to 5.26	2.27	0.056	-	-	-
III	5.05 to 19.08	9.82	<0.001	-	-	-
Ⅳ	16.68 to 62.68	32.34	<0.001	-	-	-
**pT status**						
T1		1.0 (ref.)			1.00(ref.)	
T2	1.75 to 6.33	3.33	<0.001	1.14 to 4.42	2.25	0.018
T3	3.61 to 13.45	6.97	<0.001	0.77 to 3.95	1.74	0.180
T4	11.46 to 53.87	24.84	<0.001	0.24 to 2.84	0.83	0.770
**pN status**						
N0		1.0 (ref.)			1.00(ref.)	
N1	8.26 to 23.75	14.00	<0.001	3.00 to 13.65	6.40	<0.001
**pM status**						
M0		1.0 (ref.)			1.00(ref.)	
M1	9.47 to 30.27	16.93	<0.001	1.47 to 8.12	3.45	0.004
**ALP**						
Low/Normal		1.0 (ref.)				
Elevated	0.80 to 6.10	2.22	0.123	-	-	-
**Blood platelet**	1.00 to 1.01	1.01	<0.001	0.99 to 1.00	1.00	0.579
**Fibrinogen**						
≤4.42		1.00(ref.)			1.00(ref.)	
>4.42	3.46 to 9.45	5.72	<0.001	1.00 to 3.70	1.92	0.049
**D-dimer**						
≤0.95		1.00(ref.)			1.00(ref.)	
>0.95	2.69 to 7.28	4.43	<0.001	1.07 to 2.03	1.47	0.017

Abbreviation: HR, hazard ratio; CIs, confidence intervals; BMI, body mass index; pTNM, pathologic tumor-node-metastasis; ALP, alkaline phosphatase.

**Table 3:** Univariate and multivariate analyses for variables considered for disease-free survival (Cox proportional hazard regression model)

	DFS Univariate analysis			DFS Multivariate analysis		
Characteristics	95.0% CIs	HR	P value	95.0% CIs	HR	P value
**Age (years)**	0.99 to 1.03	1.01	0.383	-	-	-
**BMI**	0.76 to 0.92	0.84	<0.001	0.80 to 0.98	0.88	0.019
**Gender**						
Male		1.00(ref.)				
Female	0.66 to 2.23	1.21	0.538	-	-	-
**Pathological types**						
clear cell carcinoma		1.00(ref.)			1.00(ref.)	
Non-clear cell carcinoma	1.03 to 3.79	1.98	0.040	0.31 to 8.42	1.63	0.556
**Fuhrman-grade**						
I		1.00(ref.)			1.00(ref.)	
II	0.41 to 3.60	1.22	0.719	0.28 to 2.69	0.88	0.823
III	0.46 to 6.32	1.70	0.430	0.17 to 2.70	0.69	0.595
Ⅳ	1.51 to 45.05	8.24	0.015	0.59 to 22.27	3.64	0.162
unknown	0.66 to 6.08	2.00	0.221	0.07 to 3.06	0.49	0.447
**pTNM stage**						
I		1.00(ref.)				
II	1.00 to 5.38	2.32	0.049	-	-	-
III	4.83 to 18.23	9.38	<0.001	-	-	-
**pT status**						
T1		1.00(ref.)			1.00(ref.)	
T2	1.34 to 5.53	2.72	0.005	1.07 to 4.80	2.27	0.031
T3	2.48 to 11.19	5.27	<0.001	0.56 to 3.69	1.45	0.436
**pN status**						
N0		1.00(ref.)			1.00(ref.)	
N1	5.92 to 23.44	11.78	<0.001	3.67 to 19.25	8.41	<0.001
**ALP**						
Low/Normal		1.00(ref.)				
Elevated	0.12 to 6.37	0.88	0.896	-	-	-
**Blood platelet**	1.00 to 1.01	1.01	0.001	0.99 to 1.00	1.00	0.971
**Fibrinogen**						
≤4.42		1.00(ref.)			1.00(ref.)	
>4.42	2.93 to 9.98	5.41	<0.001	1.62 to 6.34	3.20	<0.001
**D-dimer**						
≤0.95		1.00(ref.)			1.00(ref.)	
>0.95	1.90 to 6.32	3.47	<0.001	1.02 to 2.15	1.48	0.038

Abbreviation: HR, hazard ratio; CIs, confidence intervals; BMI, body mass index; pTNM, pathologic tumor-node-metastasis; ALP, alkaline phosphatase.

**Table 4:** Multivariate analyses for variables considered for overall survival and disease-free survival (Cox proportional hazard regression model)

	OS Multivariate analysis			DFS Multivariate analysis		
Characteristics	95.0% CIs	HR	P value	95.0% CIs	HR	P value
**BMI**	0.84 to 0.99	0.91	0.038	0.80 to 0. 0.98	0.88	0.021
**Pathological types**						
clear cell carcinoma		1.00(ref.)			1.00(ref.)	
Non-clear cell carcinoma	0.37 to 3.71	1.18	0.776	0.29 to 6.10	1.35	0.696
**Fuhrman-grade**						
I		1.00(ref.)			1.00(ref.)	
II	0.37 to 4.37	1.27	0.700	0.27 to 2.55	0.83	0.749
III	0.60 to 8.37	2.24	0.228	0.24 to 3.66	0.93	0.927
Ⅳ	1.23 to 35.30	6.60	0.027	0.82 to 29.91	4.96	0.080
unknown	0.26 to 5.91	1.24	0.784	0.11 to 3.30	0.60	0.561
**pT status**						
T1		1.0 (ref.)			1.00(ref.)	
T2	1.08 to 4.19	2.13	0.027	0.93 to 4.19	1.97	0.074
T3	0.72 to 3.82	1.66	0.226	0.46 to 3.39	1.25	0.655
T4	0.24 to 2.98	0.85	0.804	-	-	-
**pN status**						
N0		1.0 (ref.)			1.00(ref.)	
N1	3.00 to 14.24	6.54	<0.001	3.69 to 21.70	8.95	<0.001
**pM status**						
M0		1.0 (ref.)				
M1	1.72 to 9.00	3.94	0.001	-	-	-
**Blood platelet**	0.99 to 1.00	1.00	0.164	0.99 to 1.00	1.00	0.273
**Risk group**						
Low risk		1.0 (ref.)			1.0 (ref.)	
Intermediate risk	1.06 to 5.38	2.39	0.034	1.08 to 7.67	2.88	0.033
High risk	1.42 to 5.07	2.54	0.008	1.19 to 5.71	2.61	0.016

Abbreviation: HR, hazard ratio; CIs, confidence intervals; BMI, body mass index.

## Abbreviations

RCC: Renal cell cancer
OS: Overall survival
DFS: Disease-free survival
HR: Hazard ratio
CI: Confidence intervals
TNM: Tumor Node Metastasis
